# *Orostachys japonicus* exerts antipancreatic cancer activity through induction of apoptosis and cell cycle arrest in PANC‐1 cells

**DOI:** 10.1002/fsn3.1207

**Published:** 2019-09-13

**Authors:** Ji Hyun Kim, Gi Suk Nam, Sung Hyun Kim, Deok Seon Ryu, Dong Seok Lee

**Affiliations:** ^1^ Department of Smart Foods and Drugs Graduate School of Inje University Gimhae Korea; ^2^ Department of Biomedical Laboratory Science Soonchunhyang University Asan Korea

**Keywords:** apoptosis, caspases, cell cycle arrest, MAPK, *Orostachys japonicus*

## Abstract

Targeted therapy at the molecular level is important for pancreatic cancer treatment. This study looked over the anticancer activity of *Orostachys japonicus* in a human pancreatic cancer cell line, PANC‐1. An ethyl acetate fraction containing quercetin, kaempferol, and flavonol glycosides from *O. japonicus* (OJE) exhibited significant anticancer activity against the PANC‐1. OJE activated caspase‐3, caspase‐8, and caspase‐9, leading to the induction of both intrinsic and extrinsic apoptosis pathways. It also inhibited cyclin D1, cyclin B1, and cyclin‐dependent kinase 4, representing cell cycle arrest at both G1/S and G2/M phases. In addition, OJE phosphorylated MAPKs such as p38, JNK, and ERK, which are important upstream signaling factors in apoptosis and arrest of cell cycle inducing system. In conclusion, OJE effectively exerted antipancreatic cancer activity via induction of apoptosis directed by both intrinsic and extrinsic pathways and arrest of cell cycle regulated at both G1/S and G2/M stages, which is activated by MAPKs, p38, JNK, and ERK.

## 1 INTRODUCTION

Pancreatic cancer is widely known as a fatal malignant tumor with a low survival rate (Qin, Hao, Tian, Xie, & Yang, 2012), and its incidence has been increasing every year worldwide (Akimoto, Lizuka, Kanematsu, Yoshida, & Takenaga, 2015). Treatment methods for pancreatic cancer include immunotherapy, surgery, and radiation therapy, among others. However, overall therapeutic performance has not been successful because most medicinal products are combined with chemical substances that cause side effects. Thus, it is necessary to establish effective and safe therapies for pancreatic cancer that do not damage normal cells. Natural products from plant extracts are a suitable alternative because of less side effects and low resistance to other drugs.

**Figure 1:**
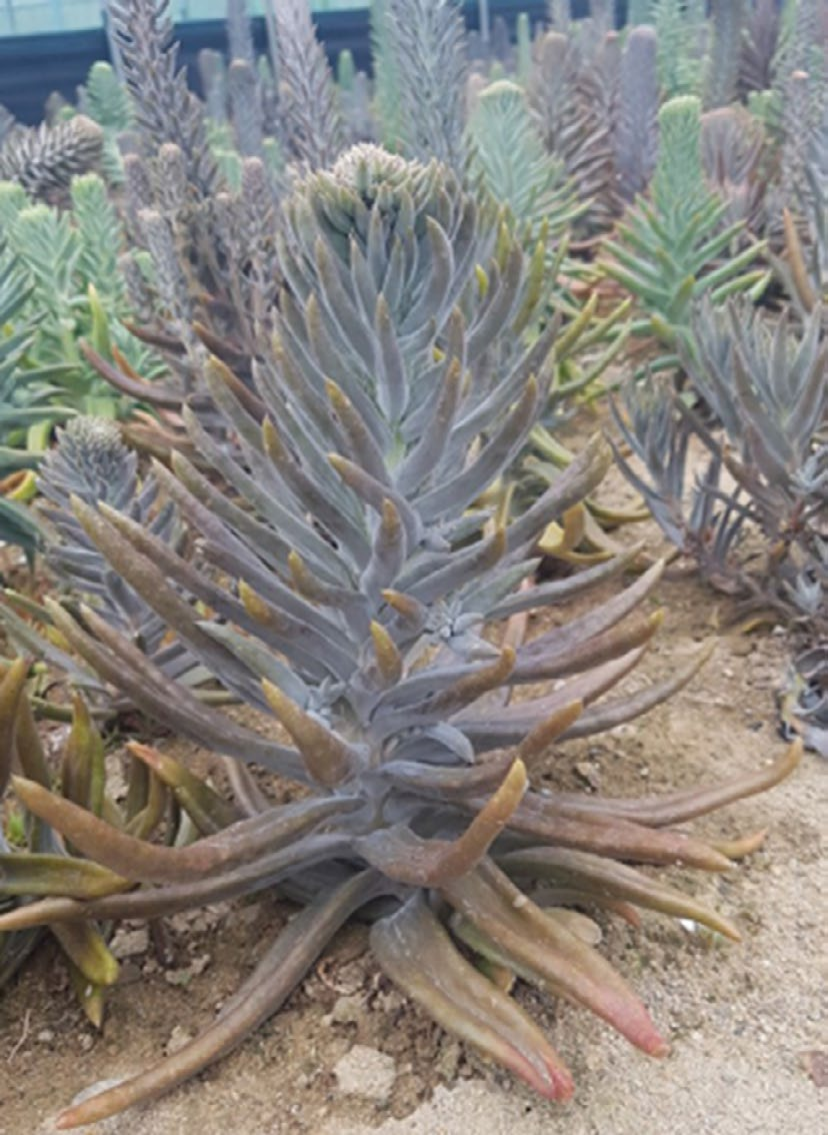
*Orostachys japonicus*

*Orostachys japonicus*, a perennial herbaceous plant belonging to the family Crassulaceae, is known as Wa‐song in Korea (Figure 1). *Orostachys japonicus* is ubiquitously found in Korea, China, Mongolia, and Japan. It is harvested from summer to autumn. Its roots are removed, dried in the sun, and used as a medicinal ingredient. The dried plant has been used in traditional Korean medicine for the treatment of hemostasis, arthritis, fever, poisoning, and hepatitis owing to its anti‐inflammatory, antifebrile, hemostatic, antidotal, and anticancer activities (Jeong, Ryu, Suk, & Lee, 2011; Kim et al., 2014; Kwon et al., 2017; Lee, Lee, Kim, Suk, et al., 2014; Lee et al., 2013; Lee, Lee, Kim, Kim, et al., 2014; Lee, Ryu, Lee, & Lee, 2012; Ryu, Lee, Kwon, & Lee, 2018; Ryu, Lee, Lee, & Lee, 2012; Yoon, Woo, & Kim, 2015). Previous studies reported that *O. japonicus* contains triterpenoids (glutinol, glutinone, friedelin, epi‐friedelanol, β‐amyrin, and taraxerone), fatty acid methyl esters, sterols (β‐sitosterol and campesterol), sterol glucosides, flavonoids (kaempferol, quercetin, and flavonoid glycosides), oxalic acid, etc (Lee et al., 2013; Park, Lim, Lee, & Young, 1994; Park, Young, Kim, Rhee, & Choi, 1991; Park, Young, Park, et al., 1991; Ryu et al., 2018).

Apoptosis is an important pathway that is triggered by both pathological and physiological conditions. Dysfunction of apoptosis leads to the development of various cancers in humans, whereas regulation of apoptosis can avoid the same. Accumulating evidence suggests that apoptosis or cell cycle arrest is closely related to anticancer therapy. In apoptosis, biochemical events lead to distinct changes in the cell, including shrinkage, blebbing, DNA fragmentation, and chromatin condensation (Kaufmann & Earnshaw, 2000) through intrinsic and extrinsic stimuli (Call, Eckhardt, & Camidge, 2008; Kacar et al., 2017; Olechowska‐jarząb, Ptak‐belowska, & Brzozowski, 2016). The intrinsic pathway functions in response to cell damage and cellular stress stimuli. Following the reception of these signals, the ratio of Bcl‐2 family members in mitochondrial outer membrane is altered, affecting cytochrome c release and resulting in activation of procaspase‐9 (Antonsson, 2004; Jiang & Wang, 2004). The extrinsic pathway is triggered by extracellular stimuli through death receptors at the cell surface, and its activation leads to the activation of procaspase‐8. The subsequent activation of intrinsic and extrinsic pathway initiates the caspase cascade, causing the activation of effector caspase‐3 (Ashkenazi, 2008; Wong, 2011).

Changes in the cellular components during the cell cycle depend on the inhibition or induction of apoptosis in cancer cells (Evan & Vousden, 2001). The cell cycle consists of four phases: G1 (Gap1), S (synthesis), G2 (Gap2) (collectively known as interphase), and M (mitosis). There are also three checkpoints: G1/S, G2/M, and metaphase. These checkpoints regulate the proper functioning of the cell cycle. CDKs are key mediators of cell cycle checkpoints and regulate the cell cycle (Williams & Stoeber, 2012). The MAPK family comprises p38 mitogen‐activated protein kinase (p38), c‐Jun N‐terminal kinase (JNK), and extracellular signal‐regulated kinase (ERK; Lunghi et al., 2017). Studies on cell death have shown that MAPKs are involved in the induction of apoptosis and are formed by various stimuli (Chen, Meyer, & Tan, 1996; Sebolt‐leopold & Herrera, 2004).

Thus, we studied the inducing effect of the ethyl acetate fraction (OJE) containing quercetin, kaempferol, and flavonol glycosides from *O. japonicus* on both apoptosis and cell cycle arrest via activation by MAPKs, p38, JNK, and ERK in a human pancreatic cancer cell line, PANC‐1.

## 2 MATERIALS AND METHODS

### 2.1 Reagents

All reagents for cell culture and Western blotting were of the highest quality or analytical grade available.

### 2.2 HPLC analysis

HPLC was performed using an Agilent 1100 series system according to the protocol of the manufacturer.

### 2.3 Cell line and culture

The PANC‐1 and CAPAN‐1 human pancreatic cancer cell lines were purchased from the Korean Cell Line Bank and cultured similarly as described in the previous study (Ryu et al., 2012). PANC‐1 cells were cultured in DMEM, and CAPAN‐1 cells were cultured in RPMI containing 10% heat‐inactivated FBS, penicillin, and streptomycin. Both cells were incubated in a cell culture dish and maintained in a humidified atmosphere containing 5% CO_2_ and 95% air at 37°C.

### 2.4 MTS assay

Inhibition of cell growth was assessed using a CellTiter 96 AQueous One Solution Cell Proliferation Assay Kit (Promega) according to the manufacturer's manual. MTS assay was carried out similarly as described previously (Ryu et al., 2018).

### 2.5 Nuclear staining assay

Nuclear staining with 4, 6‐diamidino‐2‐phenylindole (DAPI) was executed as described in earlier report (Ryu et al., 2018). Cells were visualized, and images were captured using a confocal microscope, Zeiss LSM 510 Meta.

### 2.6 Apoptosis assay

Apoptosis in PANC‐1 cells was assessed by annexin V–fluorescein isothiocyanate (annexin V‐FITC) and propidium iodide (PI) staining using an Annexin V‐FITC Apoptosis Detection Kit (BD Biosciences) according to the manufacturer's protocol. Apoptosis assay was accomplished using FACSCalibur flow cytometer (Becton Dickinson) as reported previously (Ryu et al., 2018).

### 2.7 Cell cycle analysis

Cell cycle phases were assessed by staining DNA fragments with PI using a Cell Cycle Phase Determination Kit (Cayman Chemical) according to the manufacturer's protocol. Cell cycle analysis was conducted similarly as performed previously (Ryu et al., 2018).

### 2.8 Western blotting analysis

The cells were mixed with different concentrations of OJE and harvested using a cell scraper and then resuspended on ice for 30 min, followed by removal of cell debris by centrifugation at 10,000 *g* for 10 min. Protein concentrations were measured using the bicinchoninic acid (BCA) protein assay (Pierce). Protein samples were separated on 10%–15% SDS–polyacrylamide gels through electrophoresis (Bio‐Rad) and transferred onto a polyvinylidene difluoride (PVDF) membrane. The membrane was incubated for 2 hr at room temperature with 1:5,000 dilutions of the secondary antibody (horseradish peroxidase‐conjugated goat anti‐rabbit IgG) and washed in phosphate‐buffered saline with Tween‐20 (PBST) thrice. Finally, protein concentration was measured using enhanced chemiluminescence (ECL) detection kits.

### 2.9 Statistical analysis

Statistical analysis was performed as used in the previous study (Ryu et al., 2012).

## 3 RESULTS

### 3.1 Composition of OJE from ethanol extracts of *O. japonicus*

**Figure 2:**
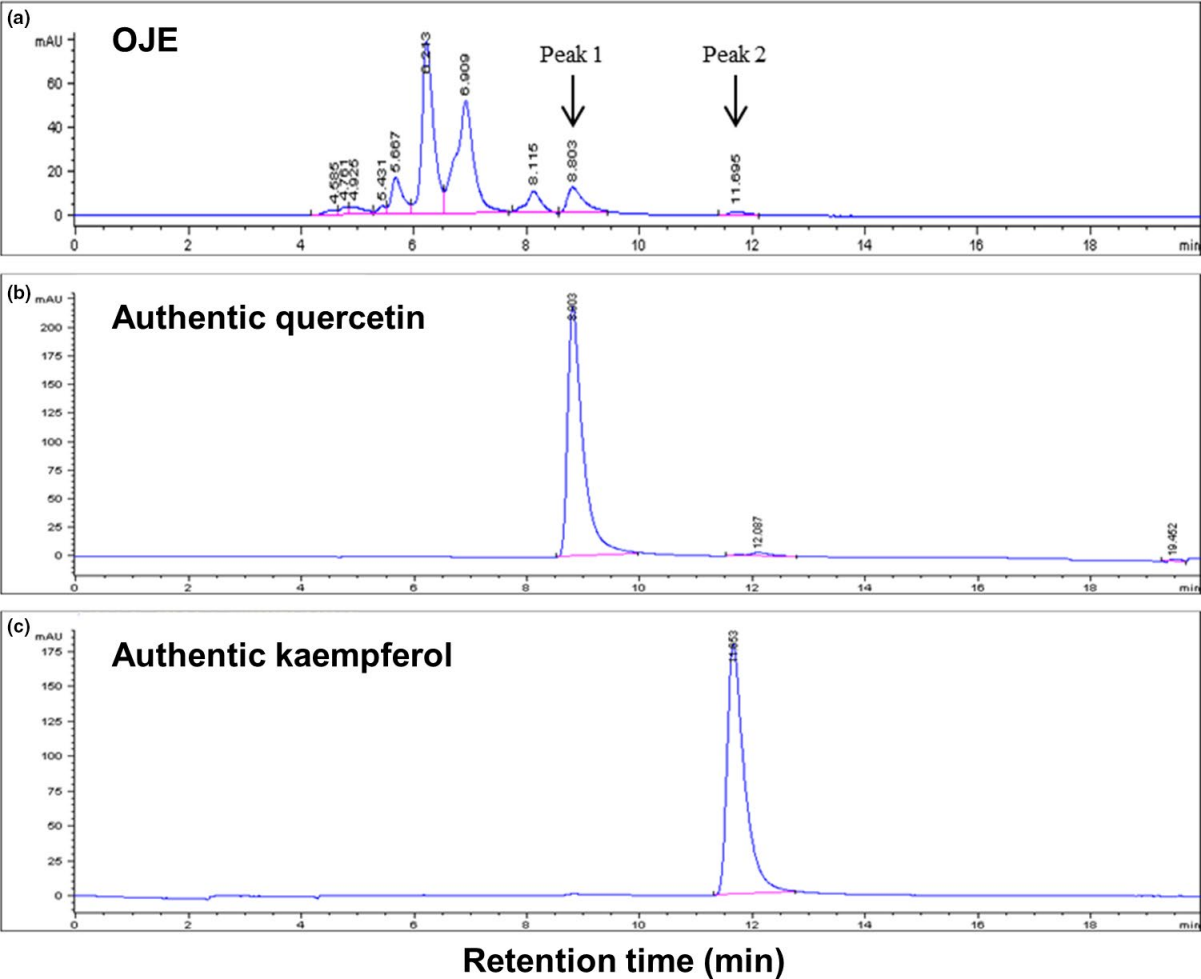
HPLC chromatograms of OJE and OJE standards—quercetin and kaempferol. Chromatograms of OJE (25 mg/ml) (a). Chromatograms of OJE standard quercetin (1,000 μg/ml) (b). Chromatograms of OJE standard kaempferol (1,000 μg/ml) (c). HPLC was performed as described in “Section 2”

OJE contains both quercetin and kaempferol as detected by HPLC in our experiment (Figure 2). As shown in Figure 2, peak 1 was observed at a retention time of 8.803 min, which was almost in accordance with the retention time (8.803 min) of quercetin (Figure 2b). Peak 2 was observed at a retention time of 11.695 min, which was similar in accordance with the retention time (11.653 min) of kaempferol (Figure 2c). Other peaks are assumed to be flavonol glycosides (C_21_), such as kaempferol‐3‐rhamnoside (afzelin), kaempferol‐3‐glucoside (astragalin), quercetin‐3‐rhamnoside (quercitrin), and quercetin‐3‐glucoside (isoquercitrin; Park, Young, Park, et al., 1991).

### 3.2 Effects of OJE on cell proliferation

**Figure 3:**
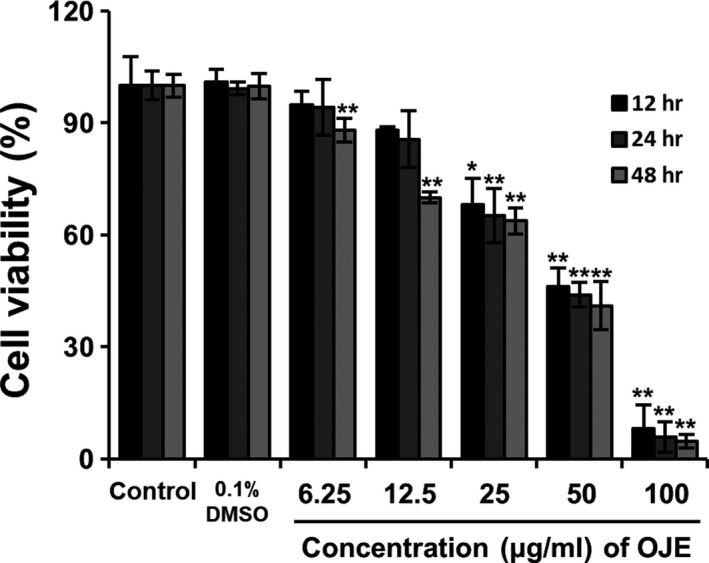
Effects of OJE on cell proliferation. PANC‐1 cell proliferation determined by a 3‐[4,5‐dimethylthiazol‐2‐yl]‐5‐[3‐carboxymethoxyphenyl]‐2‐[4‐sulfophenyl]‐2H‐tetrazolium (MTS), followed by treatment with 0.1% DMSO and different concentrations of OJE (6.25, 12.5, 25, 50, and 100 μg/ml) for 12, 24, and 48 hr. The data are expressed as mean ± *SD* (*n* = 3). *p < .05, **p < .001 versus control

Cell viability was analyzed to examine the effects of OJE by treating PANC‐1 cells with varying concentrations of OJE (6.25, 12.5, 25, 50, and 100 μg/ml) for 12, 24, and 48 hr. The effect of treatment with OJE on the proliferation of PANC‐1 cells was measured using the MTS assay. OJE significantly inhibited the proliferation of PANC‐1 cells in a concentration‐dependent manner (Figure 3).

### 3.3 Effects of OJE on apoptosis in PANC‐1 cells

**Figure 4:**
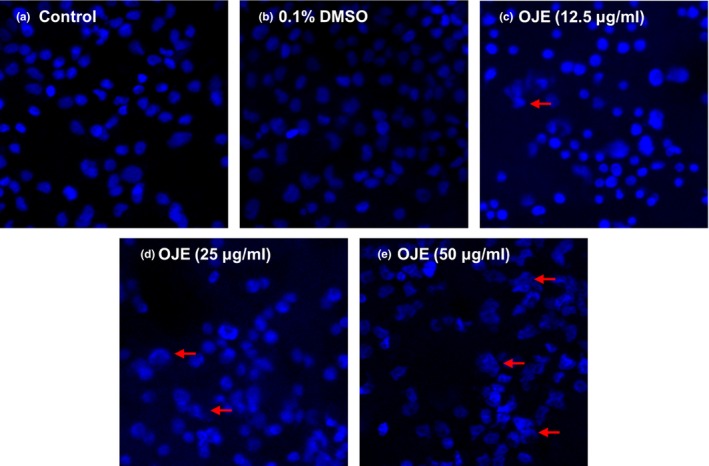
Effects of OJE on nuclear morphological changes showing features of apoptosis in PANC‐1 cells. To examine the morphological changes in PANC‐1, cells were stained with DAPI and analyzed using a confocal microscope. Control cells (a) or cells treated with 0.1% DMSO (b), 12.5 (c), 25 (d), and 50 (e) μg/ml of OJE for 12 hr were fixed. Morphological changes are indicated by red arrows

To examine whether the antipancreatic cancer effects of OJE were due to the induction of apoptosis, PANC‐1 cells were treated with OJE and apoptotic cells were observed by staining with DAPI and annexin V/PI. The results of DAPI staining demonstrated that OJE induced apoptosis of PANC‐1 cells in a dose‐related manner, indicated by chromatin condensation and nuclear fragmentation in several PANC‐1 cells (Figure 4).

**Figure 5:**
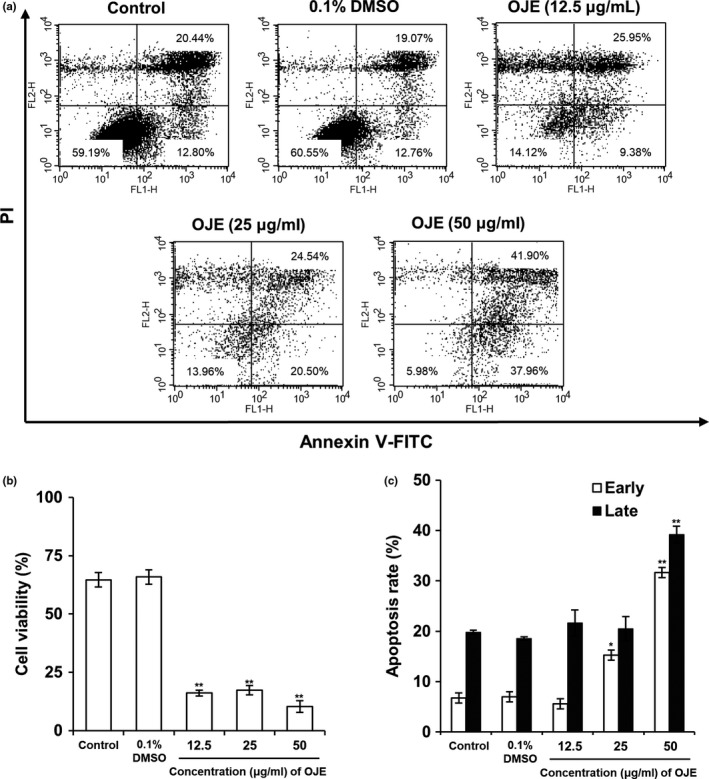
Flow cytometry analysis of the apoptotic death of PANC‐1 cells treated with different concentrations of OJE. Analysis of OJE‐induced apoptosis in PANC‐1 cells. Scatter plots represent apoptotic cell count in PANC‐1 control, PANC‐1 treated with 0.1% DMSO, or PANC‐1 treated with 12.5, 25, and 50 μg/ml OJE. PANC‐1 cells were stained with annexin V/FITC and PI and quantified by FACSCalibur flow cytometry. Apoptotic cells were calculated by counting the percentage of annexin V (+), PI (−) cells (LR), and the percentage of annexin V (+), PI (+) cells (UR). LR, lower right; UR, upper right (a). Bars indicate the percentage of viable cells (b) and percentage of early‐ and late apoptotic cells (c). The data are expressed as mean ± *SD* (*n* = 3). *p < .05, **p < .001 versus control

In flow cytometry results, the lower left quadrants in Figure 5a depict a viable normal cell group without damage. The lower right exhibits cells undergoing early apoptosis with phosphatidylserine (PS), a marker of early apoptosis, externalized to the outer layer of the membrane and DNA, another marker of late apoptosis, which was not stained by PI. The upper right displays late apoptosis. The total apoptotic rate (79.86%) of treated cells was greater than that (33.24%) of the control. Overall, these results represent that OJE treatment increased the total apoptotic rate in a dose‐dependent manner (Figure 5a,b,c).

### 3.4 Effects of OJE on regulation of apoptosis‐related proteins in PANC‐1 cells

**Figure 6:**
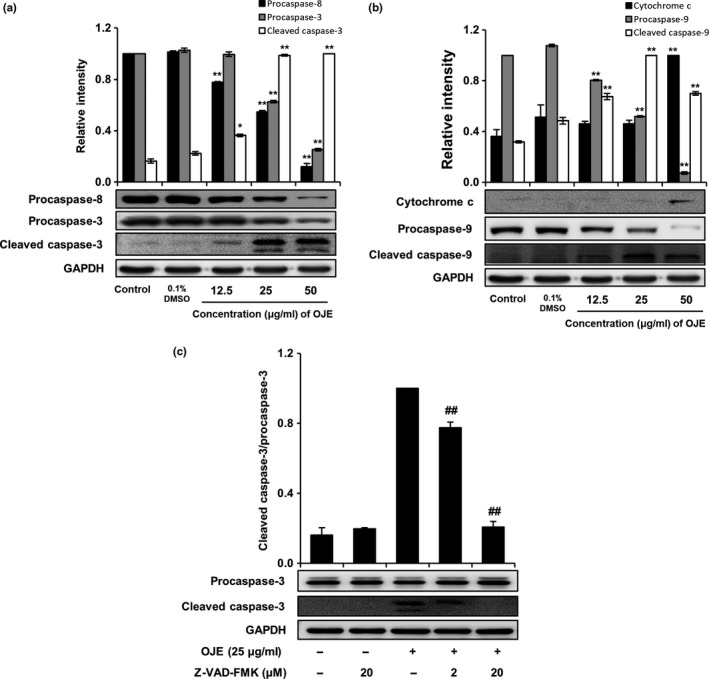
Effects of OJE on the expression of apoptosis‐related proteins in PANC‐1 cells. OJE decreased the level of procaspase‐8 and procaspase‐3, and increased the level of cleaved caspase‐3 in PANC‐1 cells in a dose‐dependent manner (a). OJE decreased the level of procaspase‐9 and increased the level of cytochrome c and cleaved procaspase‐9 in PANC‐1 cells in a dose‐dependent manner (b). Z‐VAD‐FMK decreased the level of OJE‐induced cleaved procaspase‐3 in PANC‐1 cells (c). Control cells or cells were treated with 0.1% DMSO, 12.5, 25, and 50 μg/ml of OJE for 12 hr. The expression of indicated proteins was examined by Western blotting. The data are expressed as mean ± *SD* (*n* = 3). ^*^ *p* < .05, ^**^ *p* < .001 versus control, *^##^p* < .001 versus OJE‐treated PANC‐1

Activation of caspases in cancer cells is closely associated with apoptosis. Hence, we determined whether OJE activated caspases in PANC‐1 cells. Western blotting showed that OJE effectively activated procaspases; decreased the levels of procaspase‐3, procaspase‐8, and procaspase‐9; and increased the levels of cleaved caspase‐3, cleaved caspase‐9, and cytochrome c in a concentration‐dependent manner, compared to the control in PANC‐1 cells (Figure 6a,b). On the contrary, procaspase‐3 activated by OJE was completely inhibited by Z‐VAD‐FMK (20 μM) in PANC‐1 cells (Figure 6c). This result indicates that apoptosis induction in PANC‐1 cells by OJE correlates with the cascade‐type activation of caspases.

### 3.5 Effects of OJE on cell cycle arrest in PANC‐1 cells

**Figure 7:**
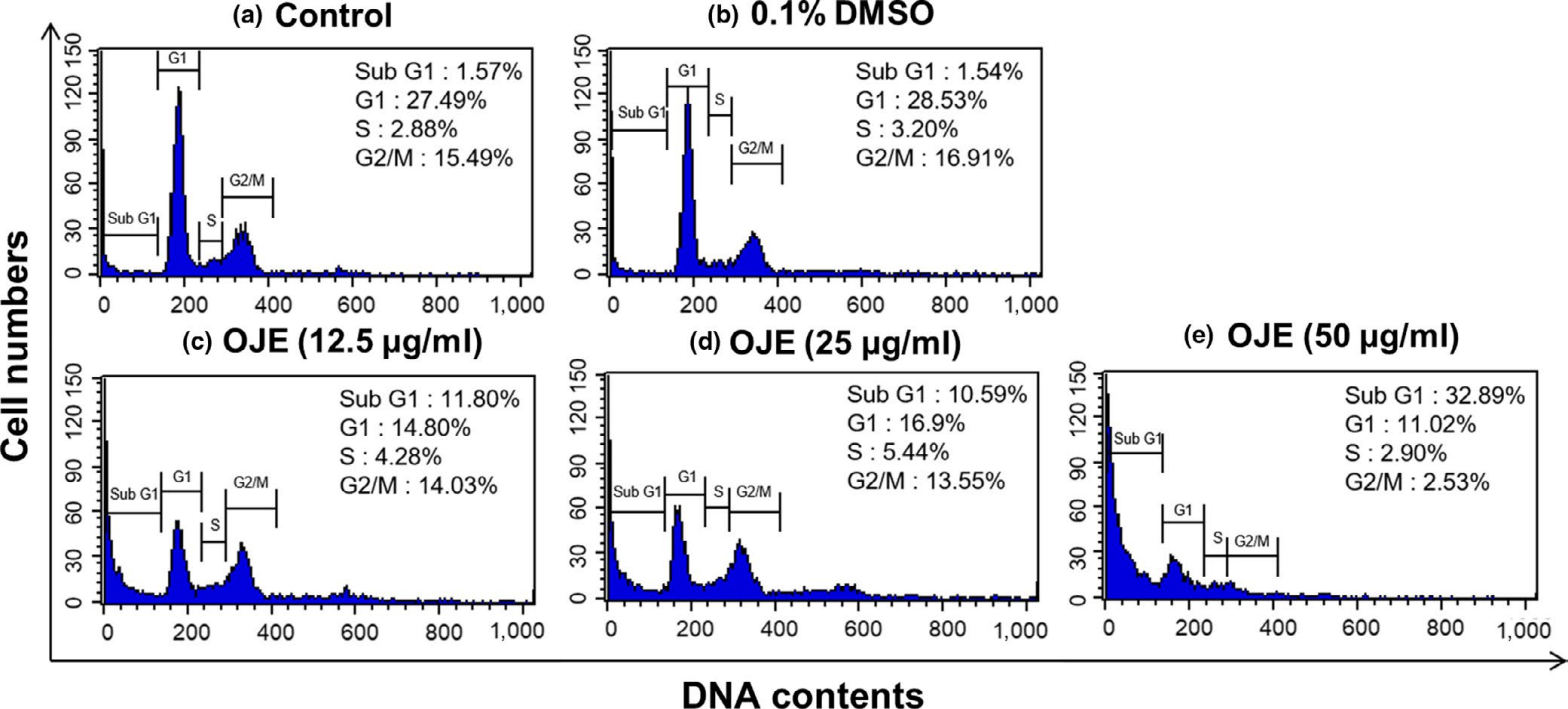
Flow cytometry analysis of cell cycle distribution of PANC‐1 cells treated with different concentrations of OJE. Histogram of PANC‐1 cells in various phases of the cell cycle, such as sub‐G1, G1, S, and G2/M. Untreated PANC‐1 cell (control) (a), cells treated with 0.1% DMSO (b), and cells treated with 12.5 (c), 25 (d), and 50 (e) μg/ml of OJE for 12 hr. Experimental data for three replicates are displayed

**Figure 8:**
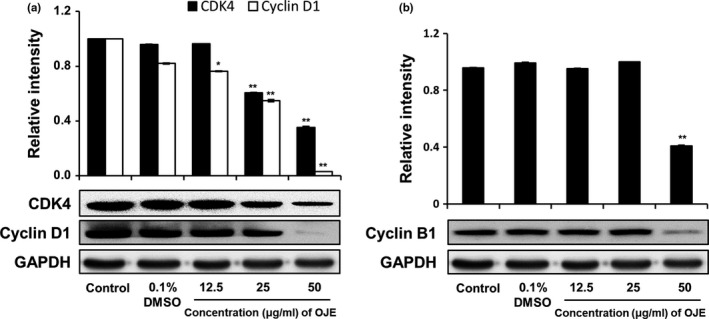
Effect of OJE treatment on protein levels of cyclin B1, cyclin D1, and CDK4 in PANC‐1 cells. PANC‐1 cells were treated with 0.1% DMSO, 12.5, 25, and 50 μg/ml of OJE for 12 hr. Protein levels of CDK4 and cyclin D1 (a) and cyclin B1 (b) were examined by Western blotting. The data are expressed as mean ± *SD* (*n* = 3). ^*^ *p* < .05, ^**^ *p* < .001 versus control

The cell cycle distribution of PANC‐1 cells was analyzed by flow cytometry. As exhibited in Figure 7, the population of control cells in the sub‐G1 phase was 1.57%, while the population of cells administered with 50 μg/ml of OJE was 32.89%. Hence, the population of cells treated with OJE increased in a concentration‐dependent manner in the sub‐G1 phase. Simultaneously, the population of the G1 and the G2/M phase cells decreased in OJE‐treated PANC‐1 cells, compared to the control (Table 1). In addition, the protein levels of CDK4, cyclin D1, and cyclin B1 measured by Western blotting decreased in a dose‐related manner (Figure 8). The decreased expression of CDK4 and cyclin D1 is believed to correlate with the decrease of cells in the G1 phase, and the decreased expression of cyclin B1 is thought to correlate with the decrease of cells in the G2/M phase.

**Table 1:** Flow cytometry analysis of cell cycle distribution of PANC‐1 cells treated with different concentration of OJE

Condition	Sub‐G1	G1	S	G2/M
Control	1.57 ± 0.45	27.49 ± 0.69	2.88 ± 0.46	15.49 ± 6.72
0.1% DMSO	1.54 ± 0.28	28.53 ± 3.29	3.20 ± 0.89	16.91 ± 7.84
OJE (12.5 μg/ml)	11.80 ± 0.30*	14.80 ± 0.92*	4.28 ± 0.08	14.03 ± 0.24
OJE (25 μg/ml)	10.59 ± 0.31*	16.9 ± 0.8*	5.44 ± 1.38	13.55 ± 0.86
OJE (50 μg/ml)	32.89 ± 0.27*	11.02 ± 2.42*	2.90 ± 0.64	2.53 ± 0.34

Note

The results are represented as percentage of total treated cells. Data are expressed as the mean ± *SD* (*n* = 3).

* *p* < .001 versus control.

### 3.6 Effects of OJE on the regulation of MAPKs in PANC‐1 cells

**Figure 9:**
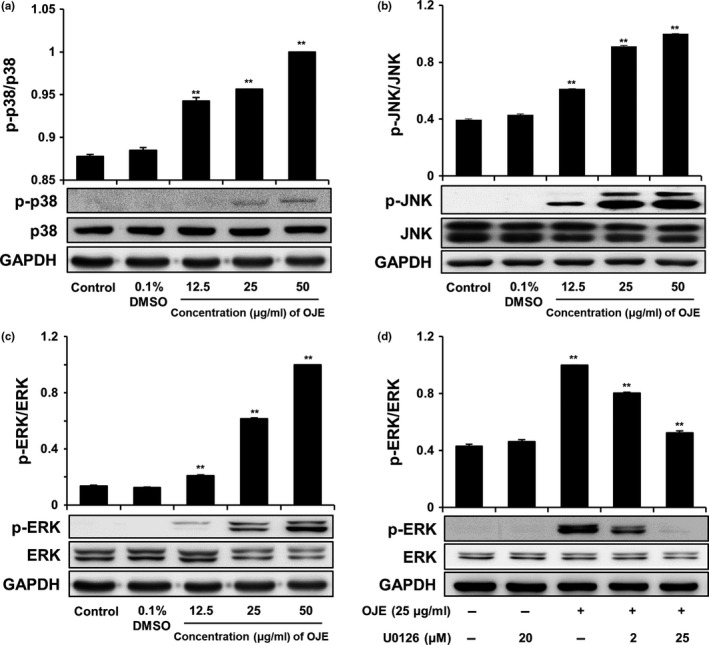
Effects of OJE on phosphorylation of MAPKs in PANC‐1 cells. OJE increased p38 phosphorylation in PANC‐1 cells in a dose‐dependent manner (a). OJE increased JNK phosphorylation in PANC‐1 cells in a dose‐dependent manner (b). OJE increased ERK phosphorylation in PANC‐1 cells in a dose‐dependent manner (c). U0126 decreased OJE‐induced ERK phosphorylation in PANC‐1 cells (d). The cells were treated with 0.1% DMSO, 12.5, 25, and 50 μg/ml of OJE for 12 hr. The expression of indicated proteins was examined by Western blotting. The data are expressed as mean ± *SD* (*n* = 3). ^**^ *p* < .001 versus control

We performed Western blotting to analyze the level of MAPK, which is known to affect upstream signaling pathways. As shown in Figure 9a,b,c, OJE raised the activation of p38, JNK, and ERK in PANC‐1 cells in a dose‐dependent manner, compared to the control. However, the levels of p38, JNK, and ERK were constant. In contrast, the OJE‐induced phosphorylation of ERK was completely inhibited by ERK inhibitor U0126 (20 μM) in PANC‐1 cells (Figure 9d). These results represent that the activation of MAPKs affecting upstream signaling pathways of apoptosis and/or arrest of cell cycle in PANC‐1 cells by OJE correlates with the phosphorylation of MAPKs, including ERK. The responsiveness of p38, JNK, and ERK to OJE implies that MAPKs are involved in the initiation of apoptosis and/or arrest of cell cycle in PANC‐1 cells.

## 4 DISCUSSION

For decades, traditional plants have been used as alternative medicines owing to their effective anti‐inflammatory, antioxidizing, antimicrobial, and anticancer activities. The reason for using natural substances is that they are often more effective, safer, and cheaper than regular medicines. In this study, we attempted to establish the molecular mechanisms of OJE‐induced apoptosis and arrest of cell cycle in PANC‐1 pancreatic cancer cells. The MTS assay results showed that OJE (exposure of 12 hr) inhibited the growth of PANC‐1 cells and displayed an inhibition concentration (IC_50_) value of 50 μg/ml (Figure 3). Induction of apoptosis has been one of the main mechanisms of anticancer therapies and is a hopeful approach for the discovery of anticancer agents (Hung & Chow, 1997; Martin & Elkon, 2004). Morphological changes observed during apoptosis, including condensation of chromatin, membrane blebbing, cellular shrinkage, and formation of apoptotic bodies, could be a sign of cell death and a convincing base for the identification of cells undergoing apoptosis. DAPI staining of OJE‐treated PANC‐1 cells showed fragmented and condensed nuclear and apoptotic bodies (Figure 4). For reference, when similar experiments were performed with other pancreatic cancer cell line, CAPAN‐1, MTS assay results of cell viability and confocal microscopy images of DAPI staining obtained from CAPAN‐1 treated with OJE looked very similar to those from PANC‐1 cells treated with OJE (data not shown). Annexin V‐FITC acts as a probe to identify apoptotic cells by binding to phosphatidylserine translocated from the cytoplasmic face to the cell surface, and the treatment of PI enables detection of early and late apoptotic cells (Mahassni & Al‐reemi, 2013). Flow cytometry indicated significant concentration‐dependent increase in apoptotic cell death and the sequential events observed during early and late apoptosis in PANC‐1 cells, especially at higher concentrations of OJE (Figure 5).

In apoptosis, the intrinsic and extrinsic pathways are categorized based on the origin of the initiating signal (inside or outside the cell). In our study, PANC‐1 cells treated with OJE raised the levels of cleaved caspase‐3 and cleaved caspase‐9, indicating the intrinsic apoptosis pathway, and lowered the level of procaspase‐8 in a dose‐related manner, indicating extrinsic apoptosis (Figure 6). Activation of caspase‐9, caspase‐8, and caspase‐3 in PANC‐1 cells suggests that apoptosis was elicited via the mitochondria‐dependent—and extrinsic mediator‐dependent—caspase cascade pathways.

Because cancer involves abnormal cell growth and proliferation, arrest of cell cycle is another effective marker of anticancer activity (Singh et al., 2004). To observe the stages of the cell cycle, PANC‐1 cells were marked with PI and quantitated using flow cytometry. The number of OJE‐treated cells in the sub‐G1 phase of the cell cycle was significantly greater (32.89%) than that in the control group (1.57%) (Figure 7). The population of the G1 and the G2/M phase cells decreased upon treatment with OJE. This result suggests the OJE induces arrest of cell cycle at both G1/S and G2/M checkpoints in PANC‐1 cells. The major mechanism of arrest of cell cycle is the regulation of the activity of protein kinase family, also known as CDK–cyclin complexes (Shackelford, Kaufmann, & Paules, 1999). Cyclin D1 is essential for inducing advancement into the G1 phase. The CDK4/cyclin D1 complex is required at the beginning of the early G1/S phase (Figure 8a). The CDK1/cyclin B1 complex leads to the advancement from G2 to M stage of the cell cycle. CDK1 is mainly activated in association with cyclin B1 during the G2/M phase. The expression of cyclin B1 was reduced when the cells were treated with OJE (Figure 8b). In addition, the MAPK pathway, including p38, JNK, and ERK, has been recognized in the process proliferation, differentiation, survival, and death of cells (Wada & Penninger, 2004). ERK is associated with both cell proliferation and cell survival. JNK and p38 are activated in response to cellular stress and cellular damage (Prasad, Vaid, & Katiyar, 2012). OJE elevated the phosphorylation of p38, JNK, and ERK in a dose‐related manner (Figure 9a,b,c). In addition, phosphorylation of ERK increased by OJE was inhibited by U0126, an ERK inhibitor (Hotokezaka et al., 2002). It is also clearly elucidated in this study that the MAPK family, including p38, JNK, and ERK, upstream signaling mediators known to affect downstream signaling pathways engaging in both apoptosis and cell cycle arrest (Cai, Chang, Becker, Bonni, & Xia, 2006; Lu & Xu, 2006; Sui et al., 2014; Taylor, Zheng, Liu, & Thompson, 2013; Wada & Penninger, 2004), is activated by OJE.

In summary, OJE effectively induces apoptosis directed by intrinsic and extrinsic caspase cascade pathway, arrest of cell cycle regulated at G1/S and G2/M stage, and activation of MAPK upstream signaling pathways, including p38, JNK, and ERK in PANC‐1 cells. Therefore, these results suggest that flavonoid‐rich OJE harboring quercetin, kaempferol, and flavonol glycosides such as afzelin, astragalin, quercitrin, and isoquercitrin from *O. japonicus* can be utilized as potential anticancer agents for patients with pancreatic cancer.

## CONFLICT OF INTEREST

The authors declare no conflict of interest.

## ETHICAL APPROVAL

This study was approved by the Korea National Institute for Bioethics Policy Public Institutional Review Board (IRB INJE NON2017‐011).
